# We View with Alarm

**Published:** 1892-10

**Authors:** 


### "WE VIEW WITH ALARM."

Let us have peace. The JOURNAL, in the language of the political platforms—that which is most heard now, "views with alarm" the heated, bitter controversy going on in Nashville, that modern Athens, where, of all places, peace on earth and good will to men should prevail, between the two medical colleges, the University of Tennessee and the Vanderbilt. The reports from there are ominous, and blood is on the moon.

The controversy grew out of the published list of matriculants in one school, which, the faculty of the other said, was in excess of the number reported to the city authorities and taxed, as the law directs, for hospital purposes; the one alleging an intentional fraud on the city and an attempt to deceive the students, while the other retorts, "Any assertion or insinuation that —— have perpetrated or intended a fraud, etc., is an infamous ——." Number one comes back that epithets are not arguments, and insults are the weapons of the guilty, and so it goes. It began at a time when students were casting around before matriculating, opened in the newspapers, and drifted into the journals, and reprints have been "like leaves in the fall, when summer was fled." Fie, fie, gentlemen (and they are calling each other "old gentlemen"), life is too short for such. It is a reflection upon our civilization that two schools can not occupy so large a city as Nashville without fighting. You each get a fine patronage; why should you quarrel over a dozen or so students, more or less ? Make peace, kiss and make friends, and let us, as before, in the language of the platform, again "point with pride" to both of you;—this viewing with alarm business is heavily done in politics; we are tired of it; it unstrings one's nerves to be so long on the stretch, and alarm is a very wearing sensation. We shall cherish the hope that the next breeze that blows southward over the classic Cumberland, will bring to our ears, not "the clash of resounding arms" (though it looked at last report as if that were the next step, and inevitable, and that they were on the Eve of battle), but the echoes of a brotherly kiss. It would be an everlasting reproach, were our veteran Confederate surgeon, or any of the Vanderbilt faculty, to go forth with the mark of Cain on his brow.

